# Microencapsulated medium-chain fatty acids as an antibiotic alternative improve intestinal immunity and microbiota composition in weaned piglets

**DOI:** 10.3389/fvets.2025.1731815

**Published:** 2026-01-09

**Authors:** Gang Zhang, Yifan Chen, Jingyi Huang, Jiashi Lang, Mingzhu Shang, Lei Shao, Zhiqiang Sun, Jinbiao Zhao

**Affiliations:** 1 State Key Laboratory of Animal Nutrition and Feeding, College of Animal Science and Technology, China Agricultural University, Beijing, China; 2 College of Animal Science and Technology, Hebei Agricultural University, Baoding, China; 3 Palmco Biotecinology Co., Ltd., Tianjin, China

**Keywords:** growth performance, gut microbiota, medium-chain fatty acids, microencapsulation, weaned piglets

## Abstract

**Objective:**

This study aimed to evaluate effects of microencapsulated medium-chain fatty acids (MCFA) on growth performance, antioxidant capacity, immune response, and gut microbiota in weaned piglets.

**Materials and methods:**

A total of 120 weaned piglets (Initial BW 6.38 ± 1.03 kg) were randomly assigned to one of three dietary treatments for a 42-day trial: a corn-soybean meal basal diet (CON), a diet supplemented with 150 mg/kg colistin sulfate (AGP), or a diet supplemented with 0.15% microencapsulated MCFA (MOA), with 5 replicates of 8 piglets per treatment.

**Results:**

During the overall period, the MOA group exhibited a higher average daily feed intake than both the CON and AGP groups (*p* < 0.05). Dietary MCFA supplementation significantly reduced (*p* < 0.05) diarrhea incidence in the first 2 weeks compared with the AGP group. On day 42, MCFA enhanced serum total antioxidant capacity compared with the AGP group and significantly lowered (*p* < 0.05) pro-inflammatory cytokines in the jejunum and colon compared with the CON group. On d 14, MCFA increased (*p* < 0.05) jejunal butyrate, lactate and jejunal and colonic total short chain fatty acids (SCFA) concentrations. Microbiota analysis revealed that MCFA modulated both jejunal and colonic communities, significantly enriching beneficial bacteria in the colon, such as *Rikenellaceae_RC9_gut_group* and *Roseburia*.

**Conclusion:**

Dietary supplementation with 0.15% microencapsulated MCFA promoted feed intake, optimized intestinal microbiota composition and metabolism, and alleviated intestinal inflammation in weaned piglets.

## Introduction

Early weaning is a critical management strategy in modern intensive pig production, employed to enhance operational efficiency through all-in/all-out systems, improve sow reproductive performance, increase farrowing room turnover, and facilitate disease control (1). However, this practice increases piglets’ susceptibility to environmental, psychological, and nutritional stressors, potentially disrupting intestinal microbiota homeostasis and impairing mucosal barrier development (2). Consequently, piglets often exhibit reduced feed intake, compromised digestibility, diarrheal disease, and in severe cases, increased mortality. In order to mitigate weaning stress in piglets, antibiotics such as colistin sulfate and chlortetracycline have been widely used as feed additives in China due to their efficacy in promoting growth and antibacterial activity (3, 4). The indiscriminate use of these antibiotics has led to drug resistance and antibiotic residues in animal products, posing risks to human health and the environment (5). In response, China implemented a comprehensive ban on antibiotic growth promoters (AGP) in animal feed in 2020 (6). This regulatory shift marks the entry of the animal production industry into a new stage and highlights an urgent need for safe, effective, and environmentally sustainable alternative strategies.

Organic acids (OA) have been widely employed in animal nutrition for decades as alternative or supplementary strategy to AGP, primarily owing to their high efficacy, environmental friendliness, and notable antibacterial activity (7, 8). Our previous studies have also demonstrated that OA can effectively reduce the intestinal pathogenic bacterial load in weaned piglets, improve gut barrier function and microbial balance, thereby alleviating diarrhea and growth retardation caused by weaning stress (9, 10). Among various OA, medium-chain fatty acids (MCFA) demonstrate distinct advantages due to their broader antimicrobial spectrum, stronger antibacterial properties, and ability to provide rapid energy (11–13). Moreover, MCFA contribute to intestinal mucosal development, enhancement of barrier function, and immune regulation (13, 14), making them particularly suitable for application during the weaning phase. However, the practical application of MCFA is often limited by their strong odor and rapid absorption in the foregut, which impair feed palatability and reduce their effectiveness. A previous study reported that dietary inclusion of blended MCFA decreased feed intake and average daily gain (ADG) in weaned pigs compared with a control group (15). Microencapsulation technology offers a solution by effectively masking the undesirable odor of MCFAs and enabling their targeted release in the distal intestine, thereby allowing more precise modulation of the hindgut microbiota and improved gut health (16). Supporting this approach, a prior study demonstrated that a microencapsulated eucalyptus MCFA product, used as an alternative to zinc oxide and antibiotics in weaned pigs, enhanced growth performance and gut health (17).

With new microencapsulation technologies for MCFA now being scaled for production, this study therefore aims to investigate the effects of microencapsulated MCFA including caprylic acid (C8:0) and capric acid (C10:0) on the gut microbiota across various intestinal segments, and its subsequent impacts on growth performance, gut microbiota community, and immune response in weaned piglets.

## Materials and methods

The experimental protocol for this study was reviewed and approved by the Institutional Animal Care and Use Committee of China Agricultural University (CAU AW51015202-1-02, Beijing, China). The experiment was performed at the Fengning Swine Research Unit of China Agricultural University (Hebei, China). The microencapsulated organic acids (MOA) used in the trial were supplied by Palmco Biotechnology Co., Ltd. (Tianjin, China), with caprylic acid (C8:0) and capric acid (C10:0) as the active constituents, together comprising 50% of the product. Briefly, a method for preparing a MOA microcapsule powder: (1) add MOA to a carbohydrate-emulsified aqueous solution and perform high-speed emulsification and shearing at a shear rate of 1,500 r/min-3,500 r/min, with the solution temperature in the tank maintained at 45 °C–70 °C; (2) homogenize the emulsified solution using a high-pressure homogenizer at a pressure of 10 MPa–40 MPa; (3) spray dry under conditions of inlet air temperature of 180 °C–220 °C and outlet air temperature of 70 °C–150 °C; (4) finally, dehumidify and dry, then collect the powder after sieving with a vibrating screen to obtain the MOA microcapsule powder.

### Animals, diets and management

A total of 120 weaned piglets [Duroc × (Landrace × Yorkshire)], with an initial age of 21 ± 2 days and an average body weight of 6.38 ± 1.03 kg, were randomly assigned to three dietary treatments. The piglets shared the same maternal genetic background (Landrace × Yorkshire dams), while the paternal line consisted of different Duroc boars. Each treatment contained 5 replicate pens, with 4 barrows and 4 gilts per pen. The dietary treatments consisted of a corn-soybean meal basal diet (CON) without antibiotics, and two experimental diets in which the basal diet was supplemented with either 150 mg/kg colistin sulfate (AGP) or 0.15% MOA. The 0.15% MOA was achieved by including 0.3% of the commercial product. All diets were formulated to meet or exceed the nutrient requirements recommended by the NRC (18) for weaned pigs (Table 1).

**Table 1:** Composition and nutrient levels of the basal diets (%, as-fed basis).

Ingredients	Content
Corn	56.45
Soybean meal, 43%	13.50
Whey powder, 3.8%	10.00
Fish meal	3.00
Soy protein concentrate	5.00
Extruded full-fat soybean	5.00
Sucrose	2.00
Soybean oil	1.30
Dicalcium phosphate	1.20
Limestone	0.75
NaCl	0.20
L-lysine HCl, 98%	0.45
DL-methionine, 98%	0.20
L-threonine, 98%	0.15
L-tryptophan	0.10
L-valine	0.20
Vitamin-mineral premix^a^	0.50
Calculated nutrient levels, %	
Digestible energy, MJ/kg	14.82
Calcium	0.83
Phosphorus	0.66
Standardized ileal digestible lysine	1.35
Standardized ileal digestible methionine	0.40
Standardized ileal digestible threonine	0.76
Standardized ileal digestible tryptophan	0.22
Analyzed nutrient levels, %	
Crude protein	18.95

a Vitamin and mineral premix provided the following per kilogram of diet: 12,000 IU vitamin A as vitamin A acetate, 2,500 IU vitamin D as vitamin D3, 30 mg vitamin E as DL-α-tocopheryl acetate, 12 μg vitamin B12, 30 mg vitamin K as menadione sodium bisulfate, 15 mg D-pantothenic acid as calcium pantothenate, 40 mg nicotinic acid, 400 mg choline as choline chloride, 30 mg Mn as manganese oxide, 90 mg Fe as iron sulfate, 80 mg Zn as zinc oxide, 10 mg Cu as copper sulphate, 0.35 mg I as ethylenediamine dihydroiodide, and 0.3 mg Se as sodium selenite.

All piglets were housed in an environmentally controlled room in pens (1.2 × 2.1 m^2^) equipped with plastic slatted floors and a mechanical ventilation system. Following weaning at 21 days of age, the piglets were transferred to the nursery facility, marking the start of a 42-day experimental period. Throughout this period, all piglets received the same basal mash diet and water *ad libitum*. The room environment was maintained at 65–75% relative humidity, with the temperature set at 28 °C for the first week and then reduced by 1 °C each week to a final temperature of 25 °C. To ensure sanitary conditions, all pens and the barn were cleaned daily.

### Sample collection

Individual body weights and pen feed consumption were measured on days 0, 14, 28, and 42, which were used to calculate ADG, average daily feed intake (ADFI), and feed to gain ratio (F:G) for each pen. Diarrhea-associated clinical signs were evaluated twice daily (morning and afternoon) by treatment-blinded technicians. Assessments were conducted visually for each piglet, supplemented by tactile examination of the perianal area to improve accuracy. A fecal scoring system was used to classify diarrhea severity according to the following criteria: 1 = hard feces; 2 = slightly soft feces; 3 = soft, partially formed feces; 4 = loose, semiliquid feces; and 5 = watery, mucous-like feces (15). A piglet was considered as experiencing a diarrheic event on a given day if the average of its two daily fecal scores exceeded 3. The incidence of diarrhea was subsequently calculated as follows:


(1) $$\begin{array}{l} \text{Diarrhea incidence}\;(\%)=\text{the total number of diarrhea piglets}\\ /(\text{total number of piglets}\;\times \;\text{total observational days})\;\times \;100 \end{array}$$

On the morning of days 14 and 42, one barrow per pen (replicate), with a body weight close to the group average, was selected after an overnight fast. A total of 8 mL of blood was drawn from the anterior vena cava into vacuum tubes. The samples were allowed to clot at room temperature for 30 min and then centrifuged at 3000 × *g* for 15 min at 4 °C (Heraeus Biofuge 22 R, Hanau, Germany). The resulting serum was aliquoted and stored at −20 °C until analysis. Following blood sampling, the pigs were euthanized by electrical stunning and exsanguination (CAU AW51015202-1-02). A complete necropsy was then performed, during which the entire intestinal tract was excised for sampling. Segments approximately 2 cm in length were collected from the mid-jejunum and mid-colon, flushed gently with saline, and fixed in 10% neutral buffered formalin. Additionally, colonic and jejunal digesta and mucosal samples were collected aseptically immediately following euthanasia. For digesta, approximately 2 g was collected from the mid-region of each intestinal segment using sterile spatulas. Mucosal samples were obtained by gently scraping the intestinal lumen with a sterile glass slide, collecting approximately 0.5 g of tissue. All collected digesta and mucosal samples were immediately snap-frozen in liquid nitrogen and subsequently stored at −80 °C until analysis.

### Analytical methods

#### Serum and intestinal mucosal immune and antioxidant indicators

Serum and mucosal concentrations of interleukin-10 (IL-10), interleukin-2 (IL-2), interleukin-1β (IL-1β), and tumour necrosis factor-α (TNF-α), as well as antioxidant parameters including malondialdehyde (MDA), superoxide dismutase (SOD), total antioxidant capacity (T-AOC), glutathione peroxidase (GSH-Px), and catalase (CAT) activities, were analyzed using commercial assay kits (H009-1, H003-1, H002, H052-1, A003-1, A001, A015-1, A005-1, A007-1, Nanjing Jiancheng Bioengineering Institute, Nanjing, China). Serum D-lactate (ml895244) and diamine oxidase (DAO, ml002413) levels were measured with porcine-specific ELISA kits (Shanghai Enzyme-linked Biotechnology Co., Ltd., Shanghai, China).

#### Intestinal morphology

The samples of intestinal tissue were fixed in 4% neutral buffered formalin for 24 h, embedded in paraffin, and sectioned at a thickness of 5 μm. Sections were then stained with hematoxylin and eosin (H&E) using standard protocols. Morphometric analysis was performed using a digital image analysis system (Leica Application Suite, Leica Microsystems, Wetzlar, Germany). Twelve well-oriented, intact villi and their associated crypts per sample were selected and measured for morphological assessment. Villus height was defined as the vertical distance from the villus tip to the crypt-villus junction, and crypt depth was measured from the crypt-villus junction to the base of the crypt.

#### Intestinal microbiota analysis

Total microbial genomic DNA was extracted from jejunal and colonic digesta using the E.Z.N.A.® Stool DNA Kit (M4015-02, Omega Bio-tek, USA) according to the manufacturer’s instructions. Subsequent library preparation, sequencing, and bioinformatic analyses were carried out as described previously (19). Briefly, the hypervariable V3-V4 region of the bacterial 16S rRNA gene was amplified with the primers 338F (ACTCCTACGGGAGGCAGCAG) and 806R (GGACTACHVGGGTWTCTAAT). The resulting amplicons were separated by agarose gel electrophoresis and purified with the AxyPrep DNA Gel Extraction Kit (Axygen Biosciences, USA). After purification, amplicons were pooled in equimolar concentrations and subjected to paired-end sequencing (2 × 250 bp) on an Illumina MiSeq platform (Illumina, USA). Raw fastq files were demultiplexed and quality-filtered using QIIME 1.17. Sequences with overlaps longer than 10 bp were merged based on their overlapping regions. Operational taxonomic units (OTU) were clustered at 97% similarity using UPARSE (version 7.1), and chimeric sequences were identified and removed with UCHIME. Taxonomic classification of OTU was performed using the RDP classifier against the Ribosomal Database Project.

#### Short-chain fatty acid analysis

Concentrations of short-chain fatty acids (SCFA) in intestinal digesta were quantified using a modified gas chromatography method (20). Briefly, approximately 0.5 g of digesta was homogenized in 8 mL of ultrapure water. The mixture was sonicated for 30 min and subsequently centrifuged at 5000 × *g* for 10 min. The supernatant was diluted 50-fold with ultrapure water, filtered through a 0.20 μm nylon membrane, and analyzed using a gas chromatography system (Agilent HP 6890 Series, Santa Clara, CA, USA) equipped with an HP 19091N-213 capillary column (30.0 m × 0.32 mm × 0.5 μm; Agilent, Santa Clara, USA) and a flame ionization detector. Nitrogen was used as the carrier gas at a constant flow rate of 2.0 mL/min. The sample (1 μL) was injected in split mode (20:1) with the injector and detector temperatures maintained at 185 °C and 210 °C, respectively.

### Statistical analysis

All data were examined for normality and outliers using the UNIVARIATE procedure in SAS 9.4 (SAS Institute Inc., Cary, NC, USA). The pen was the experimental unit for the analysis of diarrhea incidence and growth performance, which were assessed on each experimental phase, whereas the individual pig was the experimental unit for all other parameters, which were analyzed separately for each sampling day. Diarrhea incidence was compared using a chi-squared test. Other data were analyzed using the general linear model (GLM) procedure. Treatment means were compared using the LSMEANS method with Tukey’s test for multiple comparisons. For microbiome sequencing data, the relative abundance of microbial taxa and *α*-diversity indices were compared among treatment groups using the Kruskal-Wallis test for multiple comparisons. β-diversity was assessed by principal coordinates analysis (PCoA) based on the unweighted UniFrac distance. The linear discriminant analysis (LDA) effect size (LEfSe) method was employed to identify taxa with significantly differential abundance, with a logarithmic LDA score threshold set at 2.0. Statistical significance was defined as *p* < 0.05.

## Results

### Growth performance and diarrhea incidence

The diarrhea incidence and growth performance of piglets are shown in Table 2. In phase 1 (day 1–14), weaned pigs fed the MOA diet showed a higher ADFI (*p* < 0.05) compared with those fed the basal diet. Dietary supplementation with either MOA or AGP significantly reduced (*p* < 0.01) the diarrhea incidence (Equation 1). In phase 2 (day 15–28), only the AGP group continued to exhibit a lower diarrhea incidence (*p* < 0.01) compared with the CON group. During phase 3, piglets in the MOA group had a higher ADG than those in the CON group, and a higher ADFI (*p* < 0.05) than those in the AGP group. Additionally, over the entire experimental period, the ADFI in the MOA group was higher (*p* < 0.05) than that in both the CON and AGP groups.

**Table 2:** Effects of microencapsulated medium-chain fatty acids supplementation on growth performance of weaned piglets.

Item	CON	AGP	MOA	SEM	*p*-Value
Body weight, kg					
Day 1	6.36	6.38	6.40	0.26	0.284
Day 14	10.18	10.48	10.64	0.39	0.169
Day 28	16.12	16.96	16.94	0.56	0.532
Day 42	23.35	24.55	24.73	0.74	0.636
Phase 1, day 1–14					
ADG, g	275	299	303	9.99	0.172
ADFI, g	428^b^	464^ab^	497^a^	16.65	0.043
Feed: gain	1.56	1.55	1.64	0.07	0.640
Diarrhea incidence	9.71^a^	4.25^b^	5.71^b^	0.86	<0.001
Phase 2, day 15–28					
ADG, g	424	463	450	15.51	0.198
ADFI, g	642	656	673	24.50	0.729
Feed: gain	1.51	1.43	1.50	0.04	0.702
Diarrhea incidence	5.11^a^	3.69^b^	4.12^ab^	0.55	<0.001
Phase 3, day 29–42					
ADG, g	517^b^	542^ab^	556^a^	15.74	0.048
ADFI, g	977^ab^	932^b^	1073^a^	25.42	0.031
Feed: gain	1.89	1.73	1.93	0.06	0.129
Day 1–42					
ADG, g	405	434	436	11.33	0.132
ADFI, g	682^b^	684^b^	748^a^	20.05	0.038
Feed: gain	1.68	1.58	1.71	0.03	0.164

^a,b^Values with different superscripts in the same row differ significantly (*p* < 0.05; *n* = 5).

CON, basal diet without any antibiotics; AGP, CON + 150 mg/kg colistin sulphate; MOA, CON + 0.15% microencapsulated organic acids; ADG, average daily gain; ADFI, average daily feed intake.

### Serum antioxidant indicators

No significant differences were observed in serum antioxidant indicators among the treatment groups on day 14 (*p* > 0.05; Table 3). On day 42, pigs fed the MOA diet showed higher serum T-AOC (*p* < 0.05) compared with those receiving the AGP diet.

**Table 3:** Effects of microencapsulated medium-chain fatty acids supplementation on serum antioxidant capacity of weaned piglets.

Item	CON	AGP	MOA	SEM	*P*-Value
Day 14					
SOD, U/mL	81.31	76.74	85.75	8.64	0.072
GSH-PX, U/mL	321	304	322	18.35	0.936
T-AOC, U/mL	8.22	7.37	8.33	0.65	0.954
MDA, nmol/mL	4.57	4.86	4.46	0.42	0.818
Day 42					
SOD, U/mL	109.04	99.51	112.01	7.78	0.451
GSH-PX, U/mL	329	303	340	14.87	0.909
T-AOC, U/mL	10.73^ab^	9.27^b^	10.90^a^	0.46	0.031
MDA, nmol/mL	3.28	3.70	3.12	0.34	0.822

^a,b^Values with different superscripts in the same row differ significantly (*p* < 0.05; *n* = 5).

CON, basal diet without any antibiotics; AGP, CON + 150 mg/kg colistin sulphate; MOA, CON + 0.15% microencapsulated organic acids; SOD, superoxide dismutase; GSH-Px, glutathione peroxidase; T-AOC, total antioxidant capacity; MDA, malondialdehyde.

### Serum intestinal permeability biomarkers and cytokines

On day 14, the MOA group exhibited significantly lower (*p* < 0.05) DAO activity compared to the CON group (Table 4). Moreover, the IL-2 concentration in the MOA group was significantly lower than that in both the CON and AGP groups (*p* < 0.05). No differences were observed among treatments on day 42 for the evaluated parameters.

**Table 4:** Effects of microencapsulated medium-chain fatty acids supplementation on serum intestinal permeability biomarkers and cytokines of weaned piglets.

Item	CON	AGP	MOA	SEM	*p*-Value
Day 14					
D-lactate, mmol/L	2.46	2.29	2.18	0.22	0.551
DAO, U/L	5.38^a^	4.62^ab^	4.06^b^	0.31	0.018
IL-1β, ng/L	23.82	29.21	21.26	3.53	0.078
TNF-α, ng/L	43.06	47.02	43.06	6.23	0.733
IL-2, ng/L	231^a^	237^a^	197^b^	12.53	0.042
IL-10, ng/L	13.77	15.58	14.41	1.03	0.574
Day 42					
D-lactate, mmol/L	1.83	1.72	1.59	0.18	0.152
DAO, U/L	3.73	3.35	3.54	0.29	0.917
IL-1β, ng/L	20.77	21.41	15.66	2.86	0.241
TNF-α, ng/L	33.68	36.95	33.68	5.05	0.923
IL-2, ng/L	186	193	161	9.15	0.068
IL-10, ng/L	17.23	16.08	17.94	0.85	0.751

^a,b^Values with different superscripts in the same row differ significantly (*p* < 0.05; *n* = 5).

CON, basal diet without any antibiotics; AGP, CON + 150 mg/kg colistin sulphate; MOA, CON + 0.15% microencapsulated organic acids; DAO, diamine oxidase; IL-1β, interleukin-1β; TNF-α, tumour necrosis factor-α; IL-2, interleukin 2; IL-10, interleukin 10.

### Intestinal inflammatory cytokines

**Figure 1:**
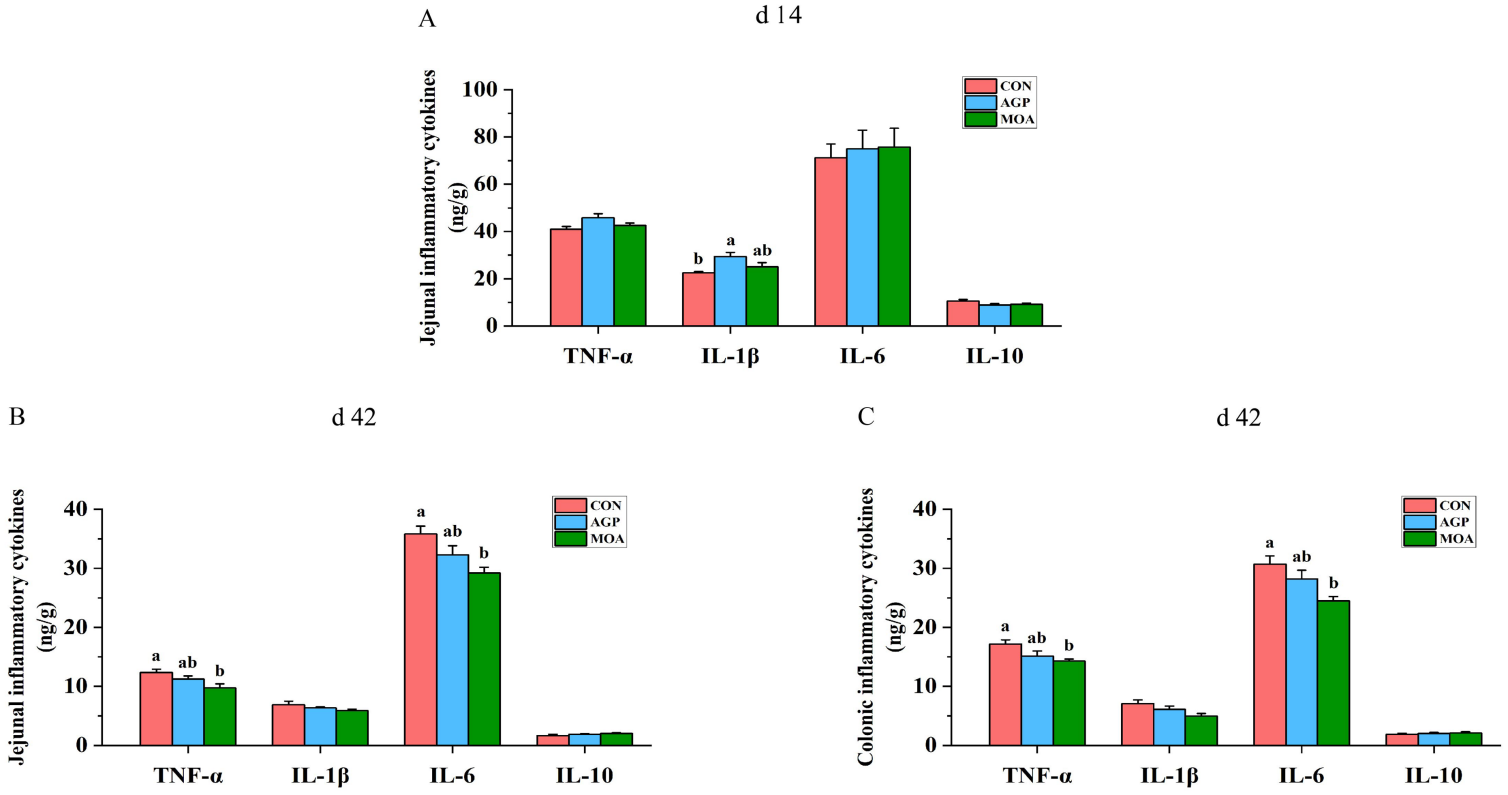
Effects of microencapsulated medium-chain fatty acids supplementation on intestinal inflammatory cytokines. **(A)** Jejunal inflammatory cytokines on day 14. **(B)** Jejunal inflammatory cytokines on day 42. **(C)** Colonic inflammatory cytokines on day 42.

On day 14, pigs fed the AGP diet exhibited significantly higher IL-1β concentration in the jejunum compared to those in the CON group (*p* < 0.05; Figure 1). On day 42, the concentrations of TNF-α and IL-6 in both the jejunum and colon were significantly lower (*p* < 0.05) in the MOA group compared with the CON group.

### Intestinal morphology

On day 14, pigs fed the MOA diet exhibited significantly greater jejunal villus height and villus height/crypt depth compared with those fed the AGP diet (*p* < 0.05; Table 5). By day 42, no significant differences in intestinal morphology indicators were observed among the three groups (*p* > 0.05).

**Table 5:** Effects of microencapsulated medium-chain fatty acids supplementation on jejunal morphology of weaned piglets.

Item	CON	AGP	MOA	SEM	*p*-Value
Day 14					
Villus height, μm	461^ab^	410^b^	492^a^	18.21	0.023
Crypt depth, μm	135	185	140	9.67	0.454
Villus height/Crypt depth	3.41^a^	2.22^b^	3.51^a^	0.34	0.021
Day 42					
Villus height, μm	554	543	590	20.06	0.729
Crypt depth, μm	215	233	251	11.41	0.431
Villus height/Crypt depth	2.58	2.33	2.35	0.22	0.812

^a,b^Values with different superscripts in the same row differ significantly (*p* < 0.05; *n* = 5).

CON, basal diet without any antibiotics; AGP, CON + 150 mg/kg colistin sulphate; MOA, CON + 0.15% microencapsulated organic acids.

### Concentration of short-chain fatty acids in intestinal digesta

The SCFA concentrations on day 14 are presented in Table 6. Compared with CON and AGP groups, pigs fed the MOA diet showed significantly higher (*p* < 0.05) concentrations of lactate and butyrate in the jejunum. Additionally, the total SCFA concentration in the jejunum and colon of the MOA group was higher (*p* < 0.05) than that in the AGP group.

**Table 6:** Effects of microencapsulated medium-chain fatty acids supplementation on short-chain fatty acids concentration in the jejunal and colonic digesta of weaned piglets on day 14.

Item	CON	AGP	MOA	SEM	*p*-Value
Jejunum					
Lactate	1.69^b^	1.43^b^	2.24^a^	0.13	0.008
Acetate	0.28	0.26	0.34	0.07	0.131
Butyrate	0.01^b^	0.01^b^	0.05^a^	0.01	< 0.001
Total SCFA	1.98^ab^	1.69^b^	2.63^a^	0.16	< 0.001
Colon					
Lactate	3.60	3.32	4.25	0.22	0.310
Acetate	2.83	2.45	3.01	0.16	0.078
Butyrate	1.26	1.34	1.52	0.11	0.192
Total SCFA	7.69^ab^	7.11^b^	8.78^a^	0.38	0.018

^a,b^Values with different superscripts in the same row differ significantly (*p* < 0.05; *n* = 5).

CON, basal diet without any antibiotics; AGP, CON + 150 mg/kg colistin sulphate; MOA, CON + 0.15% microencapsulated organic acids; SCFA, short-chain fatty acids.

### Jejunal microbiota

**Figure 2:**
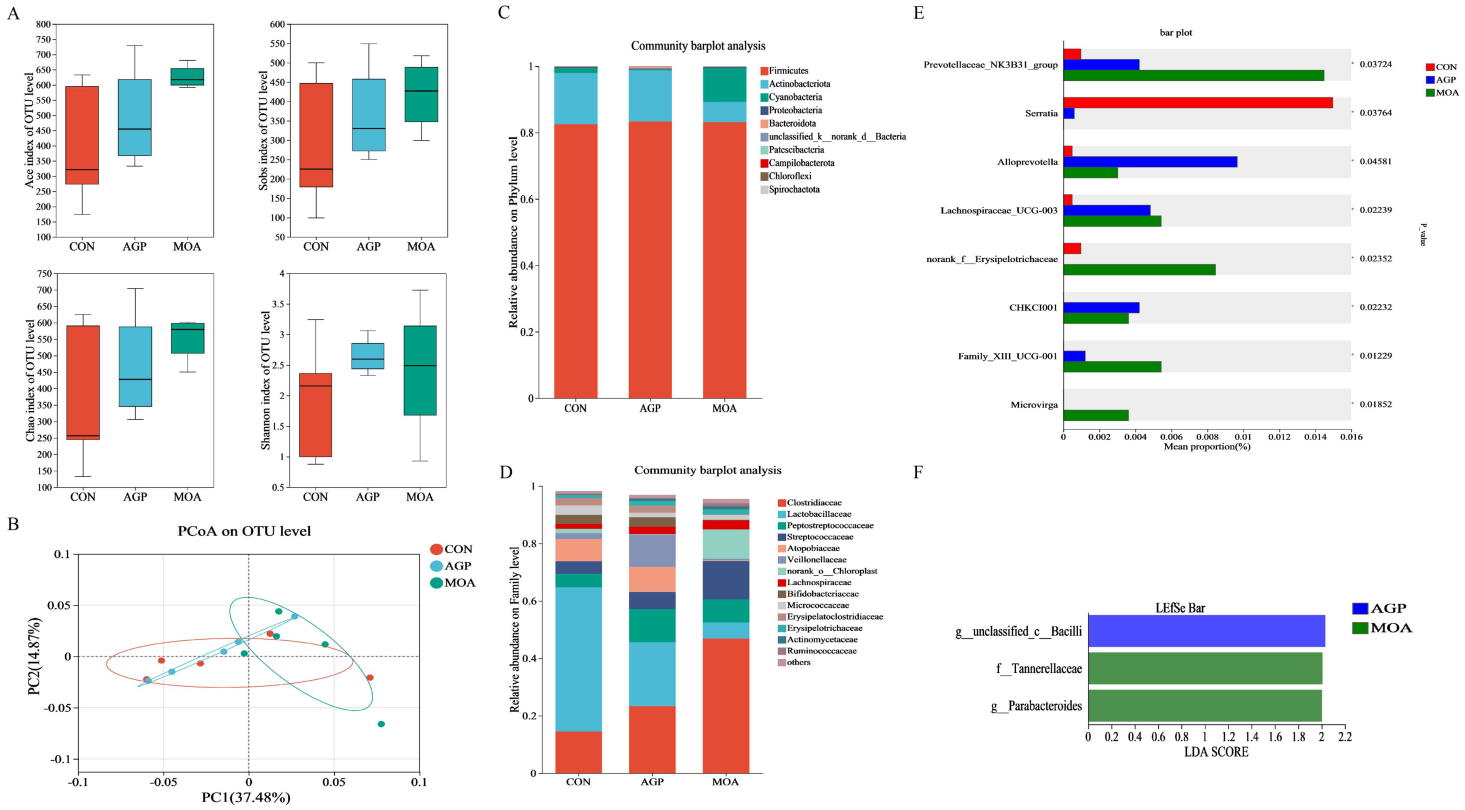
The microbial composition and structure of the jejunal digesta (*n* = 5). **(A)** The α-diversity of microbial community. **(B)** Principal coordinate analysis (PCoA) based on unweighted UniFrac distance calculated from OTU abundance matrix. **(C,D)** Microbial community compositions at phylum and family levels. **(E)** Significance testing of difference among treatments at genus level. **(F)** Bar plot of linear discriminant analysis (LDA) from phylum to genus level. CON, basal diet without any antibiotics; AGP, CON + 150 mg/kg colistin sulphate; MOA, CON + 0.15% microencapsulated organic acids.

Following sequencing, a total of 2,074,415 high-quality effective sequences were obtained after quality control and chimera removal. The average number of effective sequences per sample was 69,147 ± 2,661. The average sequence length was 416 bp, with 44.8% of sequences falling within the 421–440 bp range. These effective sequences were then clustered into operational taxonomic units (OTUs) at a 97% similarity threshold, resulting in 1,595 OTUs for subsequent species classification and analysis. The α-diversity of the jejunal microbial community, as assessed by the Ace, Sobs, Chao, and Shannon indices, showed no significant differences (*p* > 0.05) among the experimental groups (Figure 2A). Similarly, PCoA based on the unweighted UniFrac distances demonstrated no significant separation in microbial community composition across the dietary treatments (Figure 2B; *p* > 0.05). At the phylum level (Figure 2C), the CON and AGP groups were characterized by the dominance of *Firmicutes* and *Actinobacteriota*, which together constituted approximately 98% of the bacterial sequences. Specifically, *Actinobacteriota* represented 15.37 and 15.40% of the communities in CON and AGP, respectively, while the remainder consisted predominantly of *Firmicutes*. In contrast, the MOA group exhibited a markedly different composition, primarily composed of *Firmicutes* (83.14%), *Cyanobacteria* (10.23%), and *Actinobacteriota* (6.05%). At the family level (Figure 2D), the top five microbial families in the CON group were *Lactobacillaceae* (50.21%), *Clostridiaceae* (14.49%), *Atopobiaceae* (7.74%), *Peptostreptococcaceae* (4.70%), and *Streptococcaceae* (4.40%). In the AGP group, the dominant families included *Clostridiaceae* (23.39%), *Lactobacillaceae* (22.13%), *Peptostreptococcaceae* (11.61%), *Veillonellaceae* (11.09%), and *Atopobiaceae* (8.74%). The MOA group was primarily composed of *Clostridiaceae* (46.88%), *Streptococcaceae* (13.41%), *norank_o__Chloroplast* (10.22%), and *Peptostreptococcaceae* (8.02%) and *Lactobacillaceae* (5.60%).

Microbial taxa showing significant differences among groups were identified using the non-parametric Kruskal-Wallis rank-sum test (Figure 2E). Specifically, the MOA group demonstrated a significantly higher (*p* < 0.05) relative abundance of *Prevotellaceae_NK3B31_group*, *norank_f__Erysipelotrichaceae*, *Family_XIII_UCG-001*, and *Microvirga*. Conversely, *Serratia* was more abundant in the CON group, while *Alloprevotella* showed higher abundance in the AGP group. LEfSe analysis further revealed differentially enriched taxa with an LDA score >2 (Figure 2F). Specifically, the genus *unclassified_c__Bacilli* was significantly enriched in the AGP group, whereas the family *Tannerellaceae* and the genus *Parabacteroides* were most abundant in the MOA group.

### Colonic microbiota

**Figure 3:**
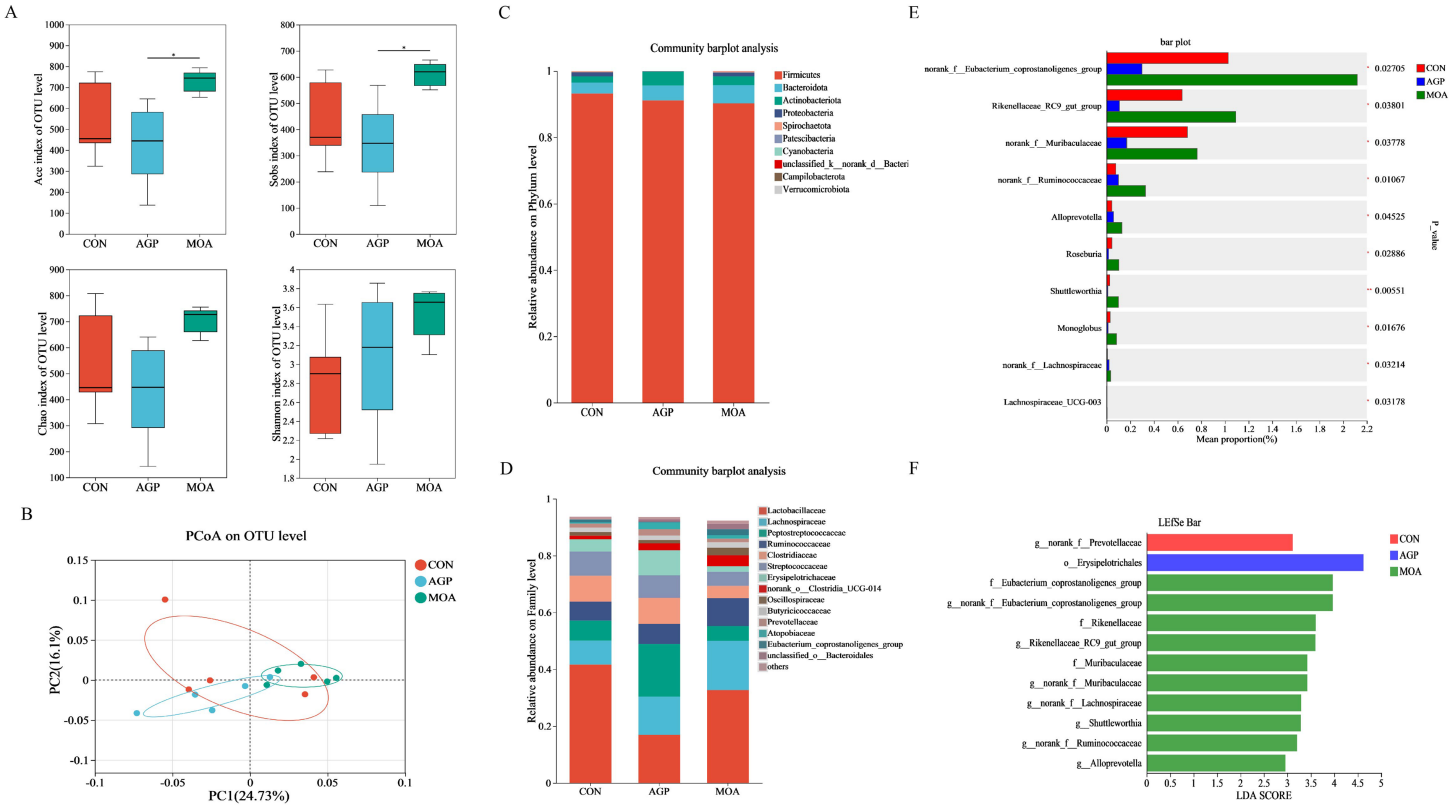
The microbial composition and structure of the colonic digesta (*n* = 5). **(A)** The α-diversity of microbial community. **(B)** Principal coordinate analysis (PCoA) based on unweighted UniFrac distance calculated from OTU abundance matrix. **(C,D)** Microbial community compositions at phylum and family levels. **(E)** Significance testing of difference among treatments at genus level. **(F)** Bar plot of linear discriminant analysis (LDA) from phylum to genus level. CON, basal diet without any antibiotics; AGP, CON + 150 mg/kg colistin sulphate; MOA, CON + 0.15% microencapsulated organic acids.

Among the α-diversity indices in colonic samples, no significant differences were observed in the Shannon and Chao indices across dietary treatments (Figure 3A). In contrast, the Ace and Sobs indices were significantly higher (*p* < 0.05) in the MOA group compared to the AGP group. The PCoA plot based on the unweighted UniFrac distances revealed no significant separation in microbial community composition among the dietary treatment groups (Figure 3B; *p* > 0.05). At the phylum level (Figure 3C), *Firmicutes* and *Bacteroideta* collectively accounted for over 95% of the sequences in all groups. At the family level (Figure 3D), the CON group was dominated by *Lactobacillaceae* (41.68%), *Clostridiaceae* (9.05%), *Streptococcaceae* (8.54%), *Lachnospiraceae* (8.41%), and *Peptostreptococcaceae* (7.05%). The AGP group showed high relative abundances of *Peptostreptococcaceae* (18.54%), *Lactobacillaceae* (16.96%), *Lachnospiraceae* (13.39%), *Clostridiaceae* (9.18%), and *Erysipelotrichaceae* (8.81%). The MOA group was primarily characterized by *Lactobacillaceae* (32.72%), *Lachnospiraceae* (17.26%), *Ruminococcaceae* (9.82%), *Peptostreptococcaceae* (5.25%), and *Streptococcaceae* (4.99%).

Multiple group comparisons revealed that the relative abundance of *Rikenellaceae_RC9_gut_group*, *norank_f__Muribaculaceae*, and *norank_f__Eubacterium_coprostanoligenes_group* was significantly higher (*p* < 0.05) in the MOA group than in the AGP group (Figure 3E). Additionally, the MOA group exhibited significantly enriched relative abundance of *norank_f__Ruminococcaceae*, *Alloprevotella*, *Roseburia*, *Shuttleworthia*, and *Monoglobus* compared to the other dietary treatment groups. The LEfSe analysis with LDA further indicated that three families (*Eubacterium_coprostanoligenes_group*, *Rikenellaceae*, and *Muribaculaceae*) and seven genera (*norank_f__Eubacterium_coprostanoligenes_group*, *Shuttleworthia*, *Rikenellaceae_RC9_gut_group*, *norank_f__Muribaculaceae*, *norank_f__Lachnospiraceae*, *norank_f__Ruminococcaceae*, and *Alloprevotella*) were enriched in the MOA group (Figure 3F). The genus *norank_f__Prevotellaceae* was significantly enriched in the CON group, while the order *Erysipelotrichales* showed significant enrichment in the AGP group.

## Discussion

Weaning is a critical phase in pig production, characterized by multiple stressors such as abrupt dietary change, physiologically and immunologically immature systems, and environmental transitions (2). A common consequence is a pronounced post-weaning reduction in feed intake, often leading to malnutrition and impaired growth rates. Dietary supplementation with MCFA, either individually or as blends, has been demonstrated to effectively mitigate these adverse effects (21). Existing research indicates that the growth-promoting efficacy of MCFA is influenced by their specific composition and inclusion level. For instance, Gebhardt et al. (22) observed that increasing the inclusion of an MCFA blend to 1.5% linearly enhanced both ADG and ADFI, while 0.5% caprylic acid alone also significantly increased ADG. Similarly, positive effects on ADG and ADFI have been reported with supplementation of a caprylic and capric acid mixture up to 2%, or a blend of caproic, caprylic and capric acid at 1.0% (23). A dose-dependent effect was further highlighted in a study where 0.55% of a combination of caproic and caprylic acids increased ADG in the first 2 weeks post-weaning, while a lower dose of 0.32% showed no significant effect (24). Research by Zentek et al. (25) showed that a 0.15% inclusion of non-encapsulated caprylic and capric acids did not improve growth performance. Notably, the present study demonstrated that microencapsulation of the same acid mixture at an identical inclusion level (0.15%) significantly increased both ADG and ADFI. These results suggest that the microencapsulation technology may enhance the bioavailability and biological efficacy of MCFA. In this study, although the overall ADFI increased, the ADG did not show a corresponding improvement. This suggests that the additional energy intake may have been preferentially utilized for enhancing intestinal development and immune function. Consequently, under the current experimental conditions, dietary supplementation with MOA could lead to an elevated feed cost per unit of weight gain.

Beyond growth performance, another major challenge during the weaning phase is the high incidence of diarrhea, and the application of MCFA also demonstrates significant potential in this regard. Our findings indicate that the microencapsulated MCFA blend exerts potent anti-diarrheal effects. This result is consistent with previous research reporting that dietary supplementation with 0.5% caproic acid or caprylic acid significantly reduced fecal scores in weaned piglets (26). Furthermore, in piglets challenged with *Escherichia coli* k88, the inclusion of 0.2% OA mixtures containing caprylic and capric acids has been demonstrated to effectively reduce the diarrhea incidence (27). Dietary supplementation with MCFA lowers the gastrointestinal pH, which enhances proteolytic enzyme activity and protein digestibility, while reducing protein fermentation in the hindgut. The resultant acidic milieu also helps inhibit the proliferation of pathogenic bacteria and optimizes the structure of the gut microbiota (7, 8). Through these mechanisms that collectively contribute to superior digestive efficiency and gut health, MCFA are effective in improving growth performance and reducing diarrhea incidence in weaned piglets. Additionally, as piglets gain body weight, intestinal maturation and microbial development progress, strengthening their resilience to environmental and pathogenic challenges (28). This largely explains the virtual absence of diarrhea observed in phase 3.

The integrity of the intestinal mucosal barrier is pivotal for preventing diarrhea, as its impairment and the consequent increase in permeability can result in substantial fluid and electrolyte efflux into the intestinal lumen, thereby inducing or exacerbating diarrhea (29). Serum DAO activity and D-lactate concentration serve as reliable biomarkers for evaluating intestinal permeability and mucosal barrier function (30). In this study, dietary supplementation with the microencapsulated MCFA blend significantly reduced serum DAO activity, whereas AGP supplementation resulted in intermediate or similar responses, with DAO values comparable to the MCFA group but not significantly different from the control group. This result aligns with the improved intestinal morphology observed in the MOA group compared to the AGP group, suggesting a stronger protective effect of MCFA on intestinal mucosal barrier function. This advantage is further supported by previous findings showing that a blend of caproic and caprylic acid glycerides more effectively improved intestinal morphology in lipopolysaccharide-challenged piglets than AGP (31). MCFA can help maintain the intestinal morphological integrity by directly supplying energy to enterocytes (14, 32). They also stimulate goblet cell proliferation and mucus secretion, which blocks the translocation of bacterial endotoxins and toxic macromolecules, preventing the reduction of tight junction protein expression, and maintaining mucosal barrier health (19, 30, 31). The lower serum IL-2 level observed in the MCFA group reflects a reduced state of systemic T-cell activation (33). This is likely attributable to the enhanced intestinal mucosal barrier, which limited the translocation of gut-derived antigens into circulation and thus diminished the persistent antigenic drive for IL-2 production.

Dietary MCFA supplementation also protected intestinal barrier integrity by suppressing pro-inflammatory factors, as evidenced by markedly reduced levels of TNF-α and IL-6 in both the jejunum and colon. This finding aligns with previous studies reporting that dietary medium-chain glycerides markedly reduced ileal IL-6 and serum TNF-α levels in weaned pigs (19, 30). Similar suppressive effects of MCFA and monoglycerides on pro-inflammatory cytokines have also been documented in sow, mice and broilers (34–36). The anti-inflammatory properties of MCFA are primarily mediated through the modulation of key signaling pathways. Specifically, MCFA and their monoglycerides downregulated the expression of toll-like receptor 4 (TLR4) and nucleotide-binding oligomerization domain protein 1, thereby inhibiting the activation of nuclear factor-κB (NF-κB) and subsequently reducing the production of TNF-α and IL-6 (36, 37). Certain MCFA, such as capric acid, have been shown to alleviate inflammatory responses and oxidative stress by directly suppressing mitogen-activated protein kinases phosphorylation and blocking NF-κB nuclear translocation (38, 39). Additionally, the higher T-AOC observed in the MCFA-supplemented group compared to the AGP group may also contribute to the anti-inflammatory effects, as excessive free radicals are known to activate the NF-κB signaling pathway (40). In line with this, Kong et al. (41) demonstrated that medium-chain glycerides increased T-AOC and SOD activity in serum and jejunum, accompanied by attenuated inflammatory responses and downregulation of the TLR4/NF-κB pathway.

The intestinal microbiota plays a vital role in nutrient digestion and absorption, intestinal epithelial development, and immune function in piglets (42, 43). A stable and diverse microbial community is fundamental to intestinal health and contributes to improved growth performance (44, 45). In the present study, dietary supplementation with microencapsulated MCFA did not affect the α-diversity of the jejunal microbiota and only led to significant enrichment of a limited number of bacterial taxa, including *Prevotellaceae_NK3B31_group*, *Family_XIII_UCG-001*, *Parabacteroides*, and *Microvirga*. These findings suggest that MCFA exert minimal influence on the overall microbial structure in the proximal gut. In contrast, more pronounced microbial shifts were observed in the hindgut following MCFA supplementation. Specifically, the colon of the MOA group exhibited numerically higher microbial richness (Ace and Sobs indices) and a greater number of differentially abundant taxa compared to the jejunum. Among these were several beneficial bacteria, such as *Alloprevotella*, *Roseburia*, *Shuttleworthia*, and *Monoglobus*, as well as certain members of *Rikenellaceae* and *Muribaculaceae*. This intestinal segment-specific response supports the hypothesis that microencapsulated MCFA function as a targeted, slow-release system, primarily regulating the distal gut where microbial density and fermentation activity are higher (13).

Consistent with our results, Li et al. (46) reported that piglets fed OA blends containing MCFA exhibited higher microbial diversity and richness in the colon compared to the AGP group. Similarly, research in sows has shown that AGP disrupted gut microbiota and decreased diversity, while MCFA supplementation promoted microbial diversity (34). The differential effects observed between the MOA and AGP groups likely stem from their distinct modes of action: AGP generally simplify the microbial community through broad spectrum inhibition, whereas MCFA help maintain a more complex and stable microbial ecosystem. This difference was clearly reflected in our study. The AGP group exhibited a microbial profile that was distinct, yet less diverse and composed of fewer dominant taxa including enrichment of *Erysipelotrichales*, a taxon associated with inflammation-related disorders in the gut (47). Conversely, the MOA group not only promoted greater microbial diversity but also enriched a broader range of beneficial bacteria in the colon, such as *Rikenellaceae_RC9_gut_group*, *Alloprevotella*, *Monoglobus*, *Roseburia, Shuttleworthia* and *Muribaculaceae*. These genera are known for their ability to degrade complex polysaccharides and produce SCFA (48–53), which correlated with the elevated colonic SCFA levels observed in our study. In the jejunum, lactate concentration was significantly higher in the MOA group and constituted the majority of total SCFA. This is noteworthy as it suggests a fermentative shift in the small intestine, which may carry important physiological and microbial implications. The increased jejunal lactate and butyrate concentrations may be associated with the elevated abundance of *norank_f__Erysipelotrichaceae*, as members of this family have been reported to produce both lactate and butyrate (54). The enrichment of *Prevotellaceae_NK3B31_group* may support butyrate production by providing succinate as a metabolic substrate for butyrate-producing bacteria (55). As the primary energy substrate for colonocytes, butyrate enhances intestinal barrier function and exerts anti-inflammatory effects (56). Collectively, the enrichment of SCFA producing bacteria in the MOA group indicates an enhanced fermentative capacity and suggests a potentially improved gut health status compared to the AGP group.

## Conclusion

Dietary supplementation with 0.15% microencapsulated MCFA promoted feed intake, optimized intestinal microbiota composition and metabolism, and alleviated intestinal inflammation in weaned piglets. As a potential antibiotic alternative, microencapsulated MCFA demonstrate distinct advantages in modulating microbial ecology and providing long-term anti-inflammatory benefits.

## Data Availability

The original contributions presented in the study are publicly available. This data can be found at: https://www.ncbi.nlm.nih.gov/bioproject/; PRJNA1387448.
